# Impact of STAS on Lung Resections for Adenocarcinoma: A Retrospective Analysis

**DOI:** 10.3390/cancers18040604

**Published:** 2026-02-12

**Authors:** Emily Belker, Katrin Hornemann, Peter Kleine, Peter Wild, Bart Vrugt, Kati Kiil, Waldemar Schreiner

**Affiliations:** 1 Division of Thoracic Surgery, University Hospital Frankfurt, Goethe University Frankfurt, 60596 Frankfurt am Main, Germany; 2 Department of Thoracic Surgery, Sana Klinikum Offenbach, 63069 Offenbach am Main, Germany; 3 Department of Pathology, Senckenberg Institute of Pathology, University Hospital Frankfurt, Goethe University Frankfurt, 60596 Frankfurt am Main, Germany; 4 Department of Pathology, Stadtspital Zürich, 8063 Zurich, Switzerland

**Keywords:** adenocarcinoma of the lung, spread through air spaces (STAS), lobectomy, sublobar resection, limited resection, histopathology, prognostic factors, overall survival, surgical oncology

## Abstract

Background: Tumor spread through air spaces (STAS) has been proposed as a histopathological marker of aggressive tumor biology in adenocarcinoma of the lung (ADCL). Its independent prognostic significance and clinical implications regarding surgical strategy remain controversial. This study evaluated clinicopathological correlates and the prognostic impact of STAS in a homogeneous cohort of resected ADCL. Methods: We retrospectively analyzed 100 patients with primary ADCL resected between 2009 and 2018. STAS was classified as absent, low (1–4 clusters), or high (≥5) by an experienced pathologist. Associations between STAS and clinical, surgical, and pathological variables were tested with univariate analyses and multivariable logistic regression. Overall survival (OS) was evaluated using Kaplan–Meier and Cox regression. Results: STAS was present in 46% of tumors and was significantly associated with a higher pathological N category (pN0-pN3; *p* = 0.005), more advanced UICC stage (*p* = 0.049), lymphovascular invasion (LVI; *p* = 0.008), and perineural invasion (PnI; *p* = 0.012). In univariate survival analysis, patients with STAS had shorter OS than patients without STAS (*p* = 0.047). After limited resection, OS did not differ (*p* = 0.864), whereas after radical anatomical resection, patients with STAS showed reduced OS (*p* = 0.034). In multivariable Cox regression analysis, STAS did not retain independent prognostic significance. Conclusions: STAS is frequent in resected ADCL and correlates with adverse pathological features and reduced OS in univariate models. In multivariate analysis, STAS did not emerge as an independent prognostic factor. These findings support the interpretation of STAS as a marker of aggressive tumor biology rather than an independent determinant of prognosis or surgical decision-making.

## Simple Summary

Lung cancer is often treated by surgically removing the tumor. However, even patients with early-stage disease can experience recurrence after surgery. One histological feature that has gained increasing attention is spread through air spaces (STAS), a pattern in which tumor cells are found beyond the main tumor within the surrounding lung tissue. In this study, we analyzed patients who underwent surgical resection for non-small cell lung cancer and examined the association between STAS and long-term survival. We found that STAS was associated with worse survival outcomes, including in patients with early-stage tumors. These findings suggest that identifying STAS after surgery may help to better estimate individual risk and support discussions about the most appropriate surgical approach. Importantly, STAS cannot yet be reliably detected before or during surgery, and its role in guiding additional treatment remains unclear. Further prospective studies are needed to clarify how STAS should be integrated into clinical decision-making.

## 1. Introduction

Lung cancer remains the leading cause of cancer-related mortality worldwide [1]. Even after complete resection with negative margins (R0), up to 30% of patients with stage I non-small cell lung cancer (NSCLC) develop recurrence, which emphasizes the persistent need for robust histopathological prognosticators to refine risk stratification and guide individualized surgical decision-making [1,2].

Tumor spread through air spaces (STAS) has emerged as a distinct invasive pattern, first described by Kadota et al. and incorporated into the 2015 WHO classification of lung tumors [2,3]. Defined as detached single cells, micropapillary clusters, or solid nests within alveolar spaces beyond the tumor edge, STAS is increasingly recognized as a marker of aggressive tumor biology. Recent pathology-focused reviews have further contextualized STAS as a distinct pattern of tumor invasion in lung adenocarcinoma, while emphasizing ongoing challenges related to its definition, quantification, and clinical interpretation [4].

In resected NSCLC, particularly ADCL, STAS is more frequently observed in tumors with solid or micropapillary-predominant histology and is associated with lymphovascular invasion (LVI) and visceral pleural invasion (VPI) [5]. Beyond ADCL, STAS has also been identified in squamous cell carcinoma, pleomorphic carcinoma, atypical carcinoid, and other neuroendocrine neoplasms [6,7,8].

Multiple studies and meta-analyses have demonstrated a significant association between STAS and decreased recurrence-free survival (RFS) and overall survival (OS). STAS also correlates with adverse prognostic factors such as male sex, lymph node involvement, higher tumor stage, and poor differentiation and has been associated with unfavorable oncologic outcomes in resected ADCL [9,10].

Despite these findings, the prognostic significance of STAS remains controversial. Its potential artifactual nature, possibly due to mechanical displacement of tumor cells during specimen processing, continues to be debated [11,12]. A substantial heterogeneity in the definition, assessment, and reporting of STAS has been described, limiting comparability across studies. Nevertheless, moderate-to-good interobserver agreement among experienced pulmonary pathologists has been reported, particularly in formalin-fixed, paraffin-embedded specimens [5].

Importantly, STAS has implications for surgical decision-making in early-stage ADCL. While lobectomy remains the standard of care, sublobar resections such as segmentectomy or wedge resection are increasingly performed in patients with limited pulmonary reserve. In high-risk histologic subtypes, particularly those with micropapillary components, sublobar resections have been associated with an increased risk of locoregional recurrence, even with negative margins [9]. In patients with STAS, a surgical margin exceeding the maximum tumor diameter has been proposed to mitigate postoperative recurrence [2]. Yet the absence of standardized criteria for quantifying and reporting STAS continues to limit consistent clinical application [5].

Recently, the International Association for the Study of Lung Cancer (IASLC) acknowledged STAS as a candidate descriptor for the forthcoming 9th edition of the Tumor–Node–Metastasis (TNM) classification, reflecting growing evidence supporting its prognostic relevance across stage I NSCLC [13].

Nevertheless, STAS has not yet been incorporated into major clinical guidelines, including those of the National Comprehensive Cancer Network (NCCN) and the European Society for Medical Oncology (ESMO), representing a gap between emerging evidence and clinical practice [14,15].

Considering the ongoing debate regarding the prognostic significance of STAS and the lack of standardized histopathological evaluation criteria, we conducted a retrospective, single-center cohort study to investigate the association between STAS and established clinicopathological features in resected ADCL. Furthermore, we evaluated the prognostic impact of a semiquantitative STAS classification with the aim of improving risk stratification and informing surgical treatment strategies.

## 2. Materials and Methods

### 2.1. Study Design and Setting

This retrospective, single-center cohort study was conducted at the Department of Thoracic Surgery, University Hospital Frankfurt, in collaboration with the Senckenberg Institute of Pathology, University Hospital Frankfurt. The study period encompassed all lung resections performed between 2009 and 2018. Clinical data were retrieved from the hospital information system (ORBIS), and histopathological data from the institutional pathology database (DCS_PATHOS).

To minimize confounding, strict eligibility criteria were applied. Of 366 patients who underwent resection for NSCLC, 100 met the final inclusion criteria. Eligible patients had histologically confirmed adenocarcinoma, R0 resection, TNM staging according to the 8th edition of the UICC classification, postoperative survival of at least 6 months, age ≥ 18 years, and available follow-up or death documentation. Patients were excluded if STAS could not be histologically assessed.

**Figure 1:**
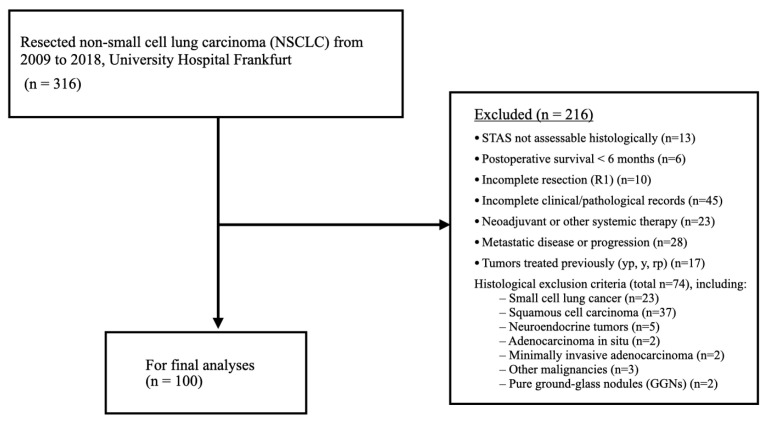
Flowchart illustrating patient selection and exclusion criteria for the final study cohort.

Additional exclusion criteria included adenocarcinoma in situ, minimally invasive adenocarcinoma, neuroendocrine tumors, squamous cell carcinoma, small cell lung cancer, pure ground-glass nodules (GGNs), other malignancies, neoadjuvant or other systemic therapy, metastatic disease at presentation, incomplete records, or incomplete resection (R1). Stage IV patients were included in cases of oligometastatic disease treated within a curative surgical concept, reflecting real-world surgical practice during the study period. Their inclusion further limits external validity and was therefore considered in the interpretation of survival analyses. A detailed overview of the patient selection process is shown in Figure 1.

### 2.2. Ethical Considerations

The study was conducted in accordance with the Declaration of Helsinki and was approved by the Ethics Committee of the University Hospital Frankfurt am Main (reference number: 2025–2615). Owing to the retrospective study design and the exclusive use of anonymized patient data, the requirement for written informed consent was waived.

### 2.3. Histopathological Evaluation of STAS

Following surgical resection, tumor specimens were immediately transferred to the Institute of Pathology to minimize cold ischemia time. All samples were formalin-fixed, paraffin-embedded (FFPE), sectioned at 3–5 µm, and routinely stained with hematoxylin and eosin (H&E) for primary histomorphological assessment. As part of the standard diagnostic work-up, additional sections were prepared where required, including frozen sections for rapid intraoperative evaluation and immunohistochemical stains (e.g., E-cadherin, vimentin) performed on separate serial sections obtained from the same FFPE blocks. All relevant slides were subsequently digitized for long-term archiving.

For this study, STAS was retrospectively assessed on whole-slide digital images by an experienced pulmonary pathologist (Dr. Bart Vrugt, University Hospital Zurich), who was blinded to all clinical information. Supporting review was conducted within a pathology team. In selected cases, an additional independent review was performed by a second pulmonary pathologist for diagnostic confirmation.

The final STAS classification was determined by the pulmonary pathology specialist. In accordance with the 2015 WHO classification, STAS was defined as the presence of single tumor cells, micropapillary clusters, or solid nests within alveolar spaces beyond the edge of the main tumor without direct continuity with the primary tumor mass. A semiquantitative grading system was used: no STAS, low STAS (1–4 detached cells or clusters), and high STAS (≥5 detached cells or clusters), as proposed by Uruga et al., due to its reproducibility and prognostic validation [16].

**Figure 2:**
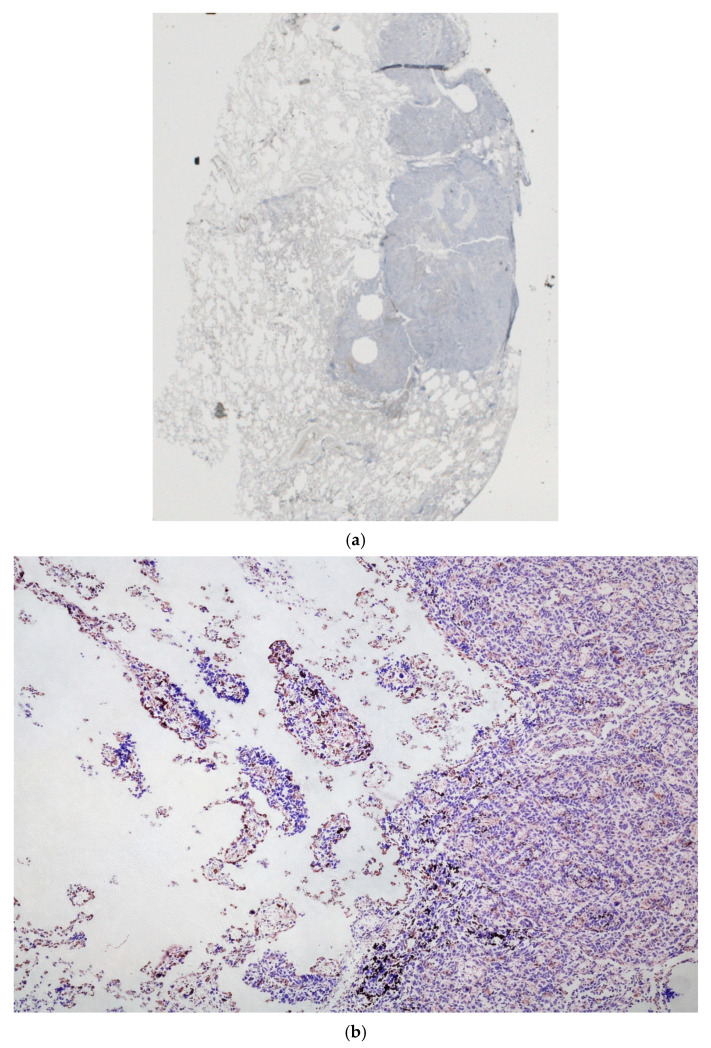
(**a**) Whole-slide overview of a lung adenocarcinoma specimen stained for E-cadherin. The image shows the entire tissue section with multiple centrally located tissue cores obtained by punching. The solid tumor component is visible on the right side of the section, while the surrounding lung parenchyma is preserved, providing spatial context for subsequent high-magnification assessment. (**b**) Representative histological examples of lung adenocarcinoma. Showing high-grade STAS, with multiple (≥5) detached tumor cell clusters within alveolar spaces beyond the main tumor edge, according to the semiquantitative grading system applied in this study [16].

This cutoff was adopted a priori and was not optimized or adjusted based on outcome data in the present cohort. No formal interobserver agreement analysis was performed, which is acknowledged as a methodological limitation. A representative example of STAS is shown in Figure 2a,b.

In most cases, STAS evaluation and grading were performed on high-resolution digital scans of routine H&E-stained sections. In a limited subset of archival specimens, original H&E slides and FFPE tissue blocks were no longer physically available. In these cases, STAS assessment was based on the best available digital material, including digitized frozen sections or immunohistochemically stained slides, provided that alveolar architecture, the tumor stroma interface, and detached tumor clusters were unequivocally preserved to allow for confident morphological evaluation. This applied to 7 of the 100 cases (7%), which were handled conservatively.

Previous studies have suggested that STAS-related morphological features may remain recognizable in non-H&E preparations under optimal conditions [2,11].

The differentiation of STAS from processing-related artifacts was based on spatial distribution, orientation of detached clusters, cytomorphologic characteristics, and reproducibility across serial sections. Resection margins were assessed after the removal of the staple line and corrected when microscopic tumor involvement was identified. LVI was considered present when either lymphatic or vascular tumor invasion was observed.

### 2.4. Clinicopathological Parameters

Data collection included patient demographics (age, sex, and tobacco exposure status), Eastern Cooperative Oncology Group (ECOG) performance status, and molecular alterations (EGFR, ALK, KRAS, and ROS1). Tumor-related variables comprised histologic subtype (acinar, papillary, solid, micropapillary, lepidic, mucinous, or cribriform), maximum tumor diameter, pathological T and N category, histologic grade, LVI, perineural invasion (PnI), VPI, and resection margin distance. Surgical variables included type of resection (lobectomy, bilobectomy, segmentectomy, wedge resection, or sleeve resection); a systematic mediastinal lymphadenectomy was performed in all patients. Postoperative complications were documented according to the Clavien–Dindo classification [17]. Follow-up variables included timing and site of recurrence, OS, and RFS. Recurrences were classified as locoregional or distant based on clinical, radiologic, or histopathologic evaluation.

### 2.5. Follow-Up

Follow-up information was obtained from hospital records and standardized outpatient assessments. Vital status and recurrence status were verified through routine clinical documentation and, when necessary, via external records. OS and RFS were defined as the time from surgery to death (OS) or the first documented recurrence (RFS). The data cut-off for follow-up was 31 December 2024. Only patients with verified survival or recurrence status were included.

The median follow-up was 50.9 months. At the last follow-up, 67 patients had died (37 with STAS and 30 without STAS), and 33 patients were alive. No loss to follow-up and no censoring occurred, as all patients had complete survival and recurrence information at the time of the data cut-off.

### 2.6. Statistical Analysis

Clinical and pathological data were extracted, anonymized, and merged into a single integrated dataset for comprehensive analysis. Statistical analyses were performed using SPSS Statistics Version 29 (IBM Corp., Armonk, NY, USA). Descriptive statistics summarized categorical variables as frequencies and percentages, and continuous variables as means ± standard deviation (SD) or medians with ranges.

Comparisons between patient groups with STAS and without STAS were conducted using the Chi-square test or Fisher’s exact test for categorical variables, and independent samples *t*-tests or Mann-Whitney U tests for continuous variables, depending on distribution. To identify factors associated with STAS, variables with *p* < 0.10 in univariate analyses were entered into a multivariable logistic regression model with STAS status as the dependent variable.

OS was analyzed using Kaplan–Meier estimates and compared using log-rank tests. Predictors of OS were evaluated using multivariable Cox proportional hazards models. Two-way interactions were assessed using partial likelihood ratio tests. Receiver operating characteristic (ROC) analyses for tumor size and age were exploratory and not included in the final multivariable model. A *p*-value < 0.05 was considered statistically significant.

Missing data were handled using a complete-case approach. All analyses were performed on the final cohort of 100 patients with complete data. No imputation was required. Model assumptions for the multivariable Cox and logistic regression models were evaluated using standard diagnostic procedures. No violations affecting model validity were identified.

All survival analyses were performed using a data cut-off of 31 December 2024, after which no additional follow-up information was incorporated. Kaplan–Meier survival curves, regression models, and ROC plots were generated using SPSS Statistics Version 29. No a priori sample size calculation was performed due to the retrospective nature of the study.

## 3. Results

### 3.1. Prevalence and Clinicopathological Characteristics of the Entire Cohort

The median age at surgery was 76.5 years (range: 41–96), and 54% of patients were male. STAS was detected in 46 of 100 cases (46%). Radical anatomical resection (lobectomy or pneumonectomy) was performed in 65% of patients, while 35% underwent limited resection (segmentectomy or wedge resection). Wedge resections comprised 27% of all procedures. A history of tobacco use was present in 90% of patients. The median tumor size was 2.7 cm (range: 0.7–10.7 cm), and the median resection margin was 1.6 cm (range: 0.2–7.2 cm). Tumors were more frequently located in the right lung (55%), with the right upper lobe being the most common site (33%).

Histologically, tumors most often showed a solid growth pattern (43%), followed by papillary (25%), acinar (23%), lepidic (5%), micropapillary (2%), and cribriform (2%) subtypes. Pathological staging revealed that 46% of patients had pT1 tumors, 21% for pT2, 21% for pT3, and 10% for pT4 disease. Lymph node metastases were absent (pN0) in 54% of cases. According to UICC staging, 25% of patients were stage IA, 8% stage IB, 15% stage II, 24% stage III, and 21% stage IV. LVI was present in 34% of tumors, and PnI in 13%. Molecular profiling revealed EGFR mutations in 23%, ALK rearrangements in 6%, ROS1 rearrangements in 22%, and KRAS mutations in 63% of tested tumors.

### 3.2. Prevalence and Clinicopathological Correlates of STAS

The patient group with STAS tumors showed a significantly higher incidence of LVI (65% vs. 35%, *p* = 0.008) and PnI (83% vs. 17%, *p* = 0.012) compared to patients without STAS. STAS was also significantly more frequent in patients undergoing radical anatomical resection compared to limited resection (54% vs. 23%, *p* = 0.006). The clinicopathological associations are summarized in Table 1.

**Figure 3:**
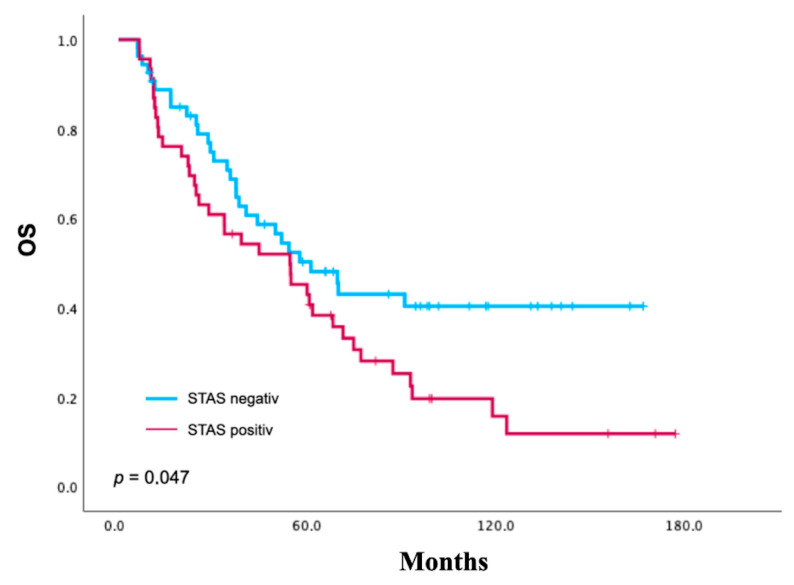
Kaplan–Meier analysis of overall survival (OS) according to STAS status in patients with resected lung adenocarcinoma (ADCL) (*n* = 100). Patients with STAS-positive tumors showed significantly reduced OS compared with patients without STAS (*p* = 0.047). Time is displayed in months. Tick marks indicate censored observations.

A significant association was observed between STAS and nodal involvement, with higher prevalence in pN1 cases compared to pN0 (67% vs. 33%, *p* = 0.005). Moreover, STAS correlated with more advanced UICC stages (*p* = 0.049), particularly stage III disease (63%). STAS distribution across T stages is shown in Figure 3.

**Figure 4:**
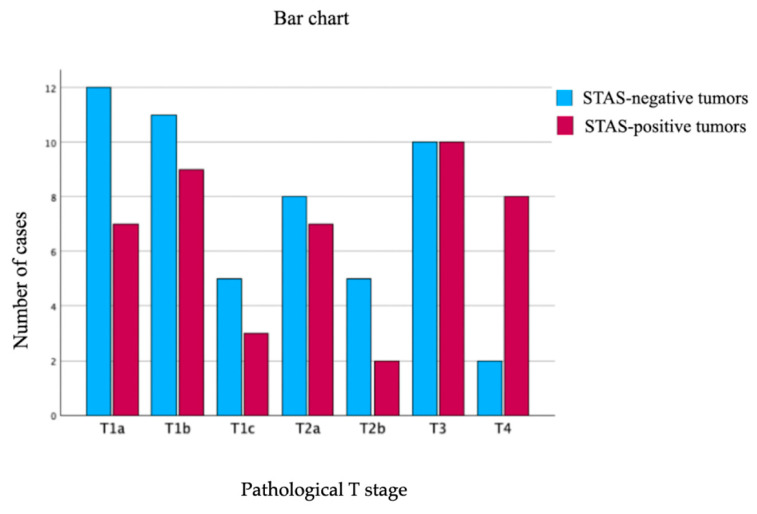
Distribution of STAS-positive and STAS-negative tumors across pathological T stages in patients with resected lung adenocarcinoma (ADCL). Bar charts show the absolute number of cases according to pathological T stage (T1a–T4), stratified by STAS status.

While STAS was present in all T stages, its relative frequency increased with advancing T category, with the highest proportions in T3 (50%) and T4 (80%) disease. Early-stage tumors (T 37%, T1b 45%) exhibited considerable STAS prevalence, indicating that STAS is not confined to large or advanced tumors. The distribution of STAS across pathological T stages is illustrated in Figure 4.

No significant difference in STAS prevalence was observed between patients aged ≤65 and >65 years (Chi-square test, *p* = 0.789).

### 3.3. Prognostic Impact of STAS

In univariate Kaplan–Meier analysis, patients with STAS had significantly shorter OS compared to those without STAS (Log-Rank χ^2^ = 3.96, df = 1, *p* = 0.047). The corresponding Kaplan–Meier curves are presented in Figure 3. Survival curves diverged early and remained separated throughout follow-up, indicating an adverse association between OS and STAS. The maximum follow-up was 170 months, with long-term censored observations primarily occurring in the patient group without STAS beyond 100 months.

In multivariable Cox regression, STAS did not remain statistically significant (HR = 0.65, 95% CI 0.321–1.309, *p* = 0.227), suggesting that its effect was attenuated by stronger prognostic variables, including nodal status (HR = 25.80, *p* = < 0.001), ECOG performance status (HR = 5.81, *p* < 0.001), and pT category (HR = 4.28, *p* = 0.025). Univariate and multivariate Cox regression analyses for overall survival are summarized in Table 2.

**Figure 5:**
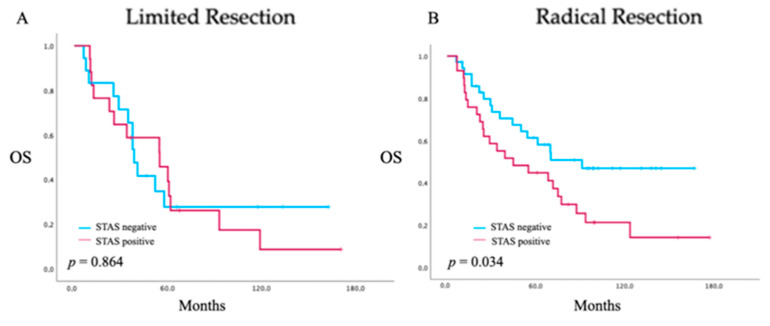
Kaplan–Meier estimates of overall survival (OS) stratified by STAS status and extent of resection in patients with resected lung adenocarcinoma (ADCL). (**A**) OS after limited resection (segmentectomy or wedge resection) in patients with and without STAS, showing no significant survival difference (*p* = 0.864). (**B**) OS after radical anatomical resection (lobectomy or pneumonectomy) stratified by STAS status, demonstrating significantly reduced OS in patients with STAS compared to those without STAS (*p* = 0.034). Time is displayed in months.

When stratified by extent of resection, no significant difference in overall survival between patients with and without STAS was observed after limited resection (*p* = 0.864), with largely overlapping and nearly parallel survival curves. In contrast, following radical anatomical resection, patients with STAS demonstrated significantly reduced overall survival compared with those without STAS (*p* = 0.034), characterized by the early and sustained separation of Kaplan–Meier curves (Figure 5). These subgroup analyses were descriptive in nature and were not adjusted for potential confounding variables. Formal interaction testing between STAS status and extent of resection was not performed due to limited sample size and insufficient statistical power, so the findings should be interpreted as exploratory only.

These findings are consistent with previous reports demonstrating an association between STAS and adverse outcomes after surgical resection, most consistently observed in sublobar resections [18].

The present subgroup analyses were descriptive in nature, and no formal interaction testing between STAS and the extent of resection was performed due to limited sample size and insufficient statistical power. Subgroup findings should be interpreted with caution. Although STAS did not emerge as an independent prognostic factor in our multivariable model, its persistent adverse association in univariate and subgroup analyses may reflect its relevance as a marker of aggressive tumor biology.

The lack of statistical significance in the multivariable model is most likely attributable to limited sample size and reduced statistical power rather than the absence of a biological effect.

Due to incomplete and heterogeneously documented recurrence data, robust statistical evaluation of RFS was not feasible. Accurate determination of recurrence timing and distinction between locoregional and distant relapse were not possible, preventing reliable conclusions about the association between STAS and RFS in this cohort.

## 4. Discussion

### 4.1. Prognostic Role of STAS in Resected NSCLC and Implications for Surgical Strategy

In the present cohort, the prevalence of STAS increased with advancing pathological T stage, while remaining detectable in early-stage tumors. This pattern is consistent with previous reports and supports the concept that STAS reflects an aggressive tumor phenotype that is not solely dependent on tumor size. The presence of STAS in early-stage disease underlines its relevance in tumors traditionally considered suitable for limited resection.

The presence of STAS was associated with an unfavorable survival pattern in patients with resected NSCLC. Kaplan–Meier survival curves diverged early and remained separated throughout follow-up, indicating an inferior prognosis in patients with STAS. These findings align with large-scale analyses, including the IASLC dataset prepared for the forthcoming 9th edition of the TNM classification, in which STAS emerged as a prognostic marker in completely resected stage I NSCLC.

Stratified survival analyses demonstrated worse overall survival in patients with STAS who underwent radical anatomical resection. While the global comparison showed a trend toward inferior survival that did not reach statistical significance (log-rank *p* = 0.080), subgroup-specific analyses revealed significantly worse overall survival for STAS-positive patients after radical anatomical resection (*p* = 0.034). However, these subgroup analyses were unadjusted and may be influenced by confounding factors such as nodal status and tumor size; therefore, no causal conclusions can be drawn from these observations. Formal interaction testing between STAS and the extent of resection was not performed due to the limited sample size and insufficient statistical power for reliable interaction modeling. Subgroup analyses should therefore be interpreted as exploratory only. In contrast, no significant survival difference was observed within the limited resection subgroup. Given the limited number of patients undergoing sublobar resection, statistical power was restricted, precluding definitive conclusions regarding prognostic neutrality in this subgroup.

The absence of a detectable prognostic effect of STAS in the limited resection subgroup may additionally reflect a floor effect related to generally poor outcomes in this population. However, limited resections are frequently performed in older patients with substantial comorbidities, and reliable cause-of-death data were not consistently available in this retrospective cohort. As a result, a clear distinction between cancer-related mortality and competing risks could not be established. Consequently, non-cancer-related mortality in this frail patient subgroup may have attenuated the observable prognostic impact of STAS. This limitation should be considered when interpreting subgroup-specific survival analyses, and no definitive conclusions regarding oncologic neutrality can be drawn from these findings.

Taken together, these findings suggest that STAS identifies tumors with unfavorable biological behavior rather than directly reflecting the adequacy of surgical margins.

Consistent with this interpretation, independent dual-center analyses of clinical T1 lung adenocarcinoma have identified STAS as an independent predictor of occult lymph node metastasis, supporting its association with early nodal dissemination despite radiologically negative nodal staging [19].

Given the retrospective nature of the available evidence and the lack of reliable preoperative or intraoperative STAS detection, STAS cannot be regarded as a stand-alone determinant of surgical strategy. From a pathological standpoint, expert commentaries have highlighted that assessment of STAS on frozen sections is unreliable for guiding intraoperative surgical decisions, owing to limited diagnostic accuracy and the risk of overtreatment [11,20,21]. STAS status may contribute to postoperative risk stratification when interpreted in conjunction with established clinicopathological risk factors.

Several retrospective surgical series have reported inferior oncologic outcomes for STAS-positive tumors when resected by limited techniques. These observations remain predominantly retrospective and may be influenced by selection bias and residual confounding and should be interpreted with caution [22,23,24]. Our findings support the interpretation of STAS as a marker of aggressive tumor biology rather than a stand-alone determinant of surgical strategy [10,16].

Large multivariable models published by the IASLC and other groups have demonstrated that STAS may rival or even exceed traditional histopathological descriptors such as VPI in prognostic relevance. In contrast, STAS did not retain independent significance in our multivariable analysis, which is likely attributable to limited sample size, collinearity with other adverse prognostic variables, and restricted statistical power, rather than the absence of biological relevance.

### 4.2. Distribution of STAS Across Tumor Stages

STAS was observed across all tumor stages in our cohort, with a substantial prevalence already in early-stage (T1) tumors. These rates exceed those reported in most prior studies, in which STAS prevalence in early-stage disease typically ranges from 15% to 35% [2,10,16,18]. STAS prevalence increased stepwise towards advanced tumor stages. The relatively high proportion of advanced-stage tumors further limits extrapolation of our findings to early-stage populations in whom STAS-guided surgical decisions would be most clinically relevant.

The comparatively high prevalence observed in our study may be partly explained by the exclusive inclusion of surgically resected tumors and by strict selection criteria applied to ensure complete clinicopathological and follow-up data, thereby enhancing internal validity. However, this approach also limits cohort size and generalizability, which must be considered when comparing prevalence estimates across studies.

### 4.3. Clinical Implications and Future Directions

Taken together with the existing literature, our results reinforce that STAS is consistently associated with adverse pathological features and reduced overall survival, even in early-stage NSCLC [2,4,8,10]. Routine reporting of STAS may therefore enhance postoperative risk stratification and support multidisciplinary discussions regarding surgical strategy, rather than serve as a directive for treatment selection. Recent multicenter studies using machine learning approaches have demonstrated promising accuracy for the preoperative prediction of STAS in small lung adenocarcinomas based on clinical and radiological parameters. Since the models are not yet validated for routine clinical implementation, they should remain investigatory [25,26]. Recent studies suggest that advanced imaging-based approaches, including radiomics and artificial intelligence, may enable preoperative estimation of STAS in the future [27].

While the prognostic relevance of STAS is increasingly supported, STAS did not independently predict survival in multivariable models in the present cohort and therefore does not justify direct or actionable therapeutic recommendations such as specific margin thresholds or adjuvant treatment modifications. Recent multicenter real-world analyses suggest that adjuvant chemotherapy does not provide a clinically meaningful survival benefit in unselected stage IA STAS-positive lung adenocarcinoma [27].

The multivariable Cox regression model showed wide confidence intervals for several covariates, particularly nodal status, indicating potential model instability. This is most likely attributable to the limited sample size relative to the number of events and possible quasi-complete separation. Consequently, the study may be underpowered to detect smaller independent effects, and the absence of independent prognostic significance for STAS should be interpreted with caution, acknowledging the potential risk of type II error.

Available evidence regarding STAS-guided systemic therapy decisions remains largely retrospective and heterogeneous [27].

In addition, recurrence-free survival could not be reliably analyzed due to incomplete documentation, which limits the prognostic scope of the present study and is explicitly acknowledged as a limitation.

Future research should focus on the standardization of STAS assessment, validation in prospective multicenter cohorts, and evaluation of whether patients with STAS benefit from modified surgical or adjuvant treatment strategies in controlled clinical trials. Advances in intraoperative assessment techniques and digital pathology may further improve the clinical applicability of STAS.

### 4.4. STAS and Current Guidelines

Despite mounting evidence supporting its prognostic relevance, STAS is not currently incorporated into risk stratification models or surgical algorithms in NCCN, ESMO, or ATS/ERS guidelines. While the IASLC has acknowledged STAS in the context of the forthcoming 9th TNM edition, the absence of prospective evidence demonstrating that STAS-guided treatment decisions improve outcomes currently precludes formal guideline recommendations. Recent multicenter studies have explored the integration of STAS into future staging systems, suggesting that STAS-positive pT1 adenocarcinomas may exhibit survival comparable to pT2a disease. Prospective validation and clinical impact analyses are still required [28].

In the long term, STAS may emerge as an additional histopathological descriptor analogous to VPI, provided its reproducibility and independent prognostic value are confirmed in well-designed prospective studies. Until such integration is achieved, individualized decision-making within multidisciplinary tumor boards remains essential.

## 5. Conclusions

In this retrospective single-center cohort of patients with resected lung adenocarcinoma, STAS was frequently observed, including in early-stage tumors, and was consistently associated with adverse pathological features and reduced overall survival. However, STAS did not retain independent prognostic significance in multivariable analysis and therefore cannot be regarded as an independent prognostic factor.

Importantly, this study does not provide evidence that STAS independently predicts survival or should guide surgical decision-making. Rather, our findings confirm previously reported associations between STAS and adverse outcomes and highlight the complexity of interpreting STAS in the context of established clinicopathological factors.

Given the retrospective design, limited sample size, and lack of reliable preoperative or intraoperative assessment, STAS should not be used as a standalone criterion for surgical strategy.

The primary contribution of this study lies in the confirmatory validation of existing evidence within a well-characterized surgical cohort and in emphasizing current methodological and interpretative limitations. Prospective, adequately powered multicenter studies are required to determine whether STAS provides independent prognostic value or actionable clinical relevance beyond established risk factors.

## Figures and Tables

**Table 1:** Clinicopathological associations and patient demographics in patients with and without tumor spread through air spaces (STAS) in resected adenocarcinoma of the lung.

Associations Between Clinicopathologic Factors and Tumor Spread Through Air Spaces (STAS)					
			STAS		
	*n*	All Patients	Absent	Present	
Variables		*n* (%)	*n* (%)	*n* (%)	*p*
Age, years	*n* = 100				0.661
Median		76.5	75.5	77.5	
Range		41–96	55–96	41–94	
	*n* = 100				0.789
≤65		10 (10)	5 (50)	5 (50)	
>65		90 (90)	49 (54)	41 (46)	
Sex	*n* = 100				0.459
Women		46 (46)	23 (50)	23(50)	
Men		54 (54)	31 (57)	23 (43)	
Tobacco exposure	*n* = 78				0.166
PNSH		8 (10)	6 (75)	2 (25)	
PESH		70 (90)	38 (54)	32 (46)	
Surgery					
Limited VS radical	*n* = 100				0.705
Limited resection		35 (35)	18 (51)	17 (49)	
Radical resection		65 (65)	36 (55)	29 (45)	
Operation method	*n* = 100				0.198
Anatomical		72 (72)	36 (50)	36	
Non-anatomical		28 (28)	18	10	
Surgical approach	*n* = 100				**0.006**
Minimally invasive		26 (26)	20 (77)	6 (23)	
Invasive		74 (74)	34 (46)	40 (54)	
Tumor morphology					
Tumor size, cm	*n* = 100				0.14
≤3 cm		60 (60)	36 (60)	24 (40)	
>3 cm		40 (40)	18 (45)	22 (55)	
Tumor size, cm	*n* = 100				0.105
≤1 cm		3 (3)	3 (100)	0 (0)	
>1 cm		97 (97)	51 (53)	46 (47)	
Tumor margin, cm	*n* = 80				0.443
Mean		1.6	1.5	1.9	
Range		0.2–7.2	0.2–7.2	0.2–5	
Tumor side	*n* = 100				0.6
Left		45 (45)	23 (51.1)	22 (48.9)	
Right		55 (55)	31 (56)	24 (44)	
Tumor site	*n* = 91				0.856
LLL		20 (22)	9 (45)	11 (55)	
LUL		19 (21)	12 (63)	7 (37)	
RLL		13 (14)	7 (54)	6 (46)	
RML		9 (10)	5 (56)	4 (44)	
RUL		30 (33)	17 (57)	13 (43)	
Tumor histology	*n* = 100				0.767
Acinar		23 (23)	12 (52)	11 (48)	
Papillary		25 (25)	14 (56)	11 (44)	
Micropapillary		2 (2)	0 (0)	2 (100)	
Solid		43 (43)	24 (56)	19 (44)	
Lepidic		5 (5)	3 (60)	2 (40)	
Cribriform		2 (2)	1 (50)	1 (50)	
Pathological stage					
pT stage classification	*n* = 99				0.107
pT1		47 (48)	28 (60)	19 (40)	
pT2		21 (21)	13 (62)	8 (38)	
pT3		21 (21)	10 (48)	11 (52)	
pT4		10 (10)	2 (20)	8 (80)	
pN stage classification	*n* = 100				**0.005**
pN0		54 (54)	36 (67)	18 (33)	
pN1		18 (18)	10 (56)	8 (44)	
pN2		22 (22)	17 (77)	5 (23)	
pN3		3 (3)	2 (67)	1 (33)	
pNX		3 (3)	1 (33)	2 (67)	
WHO stage	*n* = 100				0.164
I		0 (0)	0 (0)	0 (0)	
II		40 (40)	25 (63)	15 (37)	
III		60 (60)	29 (48)	31 (52)	
IV		0 (0)	0 (0)	0 (0)	
UICC stage	*n* = 100				**0.049**
0		7 (7)	1 (10)	6 (90)	
IA		25 (25)	18 (72)	7 (28)	
IB		8 (8)	5 (63)	3 (37)	
II		15 (15)	8 (53)	7 (47)	
III		24 (24)	9 (38)	15 (62)	
IV		21 (21)	13 (62)	8 (38)	
Lymphatic invasion	*n* = 99				**0.008**
Absent		65 (66)	41 (63)	24 (37)	
Present		34 (34)	12 (35)	22 (65)	
Vascular invasion	*n* = 92				0.746
Absent		80 (87)	44 (55)	36 (45)	
Present		12 (13)	6 (50)	6 (50)	
Perineural invasion	*n* = 91				**0.012**
Absent		79 (87)	44 (56)	35 (44)	
Present		12 (13)	2 (17)	10 (83)	
Biomarker					
EGFR mutation	*n* = 70				0.961
Absent		54 (77)	30 (56)	24 (44)	
Present		16 (23)	9 (56)	7 (44)	
ALK rearrangement	*n* = 32				0.120
Absent		30 (94)	17 (57)	13 (43)	
Present		2 (6)	0 (0)	2 (100)	
ROS1 rearrangement	*n* = 23				0.964
Absent		18 (78)	11 (61)	7 (39)	
Present		5 (22)	3 (60)	2 (40)	
KRAS mutation	*n* = 16				0.302
Absent		6 (38)	2 (33)	4 (67)	
Present		10 (62)	6 (60)	4 (40)	

Significant *p*-values are shown in bold. *p*-values were calculated using the Chi-square test or Fisher’s exact test for categorical variables, and the Mann–Whitney U test for continuous variables. Abbreviations: STAS—tumor spread through air spaces; LLL—left lower lobe; LUL—left upper lobe; RUL—right upper lobe; RML—right middle lobe; RLL—right lower lobe; PNSH—person with no smoking history; PESH—person with ever smoking history.

**Table 2:** Univariate and multivariate Cox proportional hazards analyses of overall survival in patients with resected lung adenocarcinoma, stratified by STAS status.

Univariate and Multivariate Analysis of Overall Survival in Comparison of Patient Groups with and Without STAS							
		Univariate Analysis			Multivariate Analysis		
Characteristics	Reference	HR	95% CI	*p* Value	HR	95% CI	*p* Value
Age	≤65	2.33	0.84–6.48	0.106	7.89	1.95–31.91	0.004
STAS	Negative	1.26	0.76–2.10	0.365	0.65	0.32–1.31	0.227
pT stage	T1	1.20	0.99–1.27	0.085	4.28	1.20–15.28	0.025
Nodal status	N0	1.61	1.27–2.05	<0.001	25.80	6.47–102.80	<0.001
Lymphatic invasion	L0	2.68	1.60–4.49	<0.001	0.78	0.28–2.17	0.639
Vascular invasion	V	2.10	1.05–4.17	0.037	3.74	1.37–10.21	0.010
UICC stage I-IV	I	1.50	1.21–1.88	<0.001	0.10	0.3–0.42	0.001
Limited vs. radical resection	Radical resection	0.64	0.38–1.07	0.086	0.29	0.14–0.60	<0.001
ECOG	0	3.24	2.05–5.06	<0.001	5.81	2.16–15.60	<0.001

HR, hazard ratio; CI, confidence interval; STAS, spread through air spaces.

## Data Availability

The data presented in this study are not publicly available due to ethical and data protection restrictions. The datasets contain sensitive patient information and were analyzed retrospectively in accordance with institutional and ethical regulations.

## Abbreviations

The following abbreviations are used in this manuscript:

ADCL	Adenocarcinoma of the lung
ALK	Anaplastic lymphoma kinase
ATS	American Thoracic Society
CI	Confidence interval
DCS_PATHOS	Digital pathology database (institutional)
ECOG	Eastern Cooperative Oncology Group
EGFR	Epidermal growth factor receptor
ESMO	European Society for Medical Oncology
FFPE	Formalin-fixed, paraffin-embedded
GGN	Ground-glass nodule
H&E	Hematoxylin and eosin
HR	Hazard ratio
IASLC	International Association for the Study of Lung Cancer
KRAS	Kirsten rat sarcoma viral oncogene homolog
LVI	Lymphovascular invasion
NCCN	National Comprehensive Cancer Network
NSCLC	Non-small cell lung cancer
ORBIS	Hospital information system
OS	Overall survival
PnI	Perineural invasion
R0	Complete resection with negative margins
R1	Incomplete resection with microscopic residual tumor
RFS	Recurrence-free survival
ROC	Receiver operating characteristic
ROS1	ROS proto-oncogene 1, receptor tyrosine kinase
SD	Standard deviation
SPSS	Statistical Package for the Social Sciences
STAS	Spread through air spaces
TNM	Tumor–Node–Metastasis
UICC	Union for International Cancer Control
VPI	Visceral pleural invasion
WHO	World Health Organization
